# Alveolar Echinococcosis in 11-Month-Old Dog—Clinical Case

**DOI:** 10.3390/pathogens14050450

**Published:** 2025-05-02

**Authors:** Zuzana Šufliarska, Štefan Tóth, Michaela Gentil, Filip Humeník

**Affiliations:** 1 PET Point Veterinary Clinic, 984 01 Lučenec, Slovakia; 2 Department of Morphological Science, University of Veterinary Medicine and Pharmacy in Košice, 041 81 Košice, Slovakia; 3 Department of Histology and Embryology, University of Pavol Jozef Šafárik, 041 80 Košice, Slovakia; 4 Laboklin GmbH & Co. KG, Steubenstr. 4, 97688 Bad Kissingen, Germany

**Keywords:** *alveolar echinococcosis*, *Echinococcus multilocularis*, parasitic zoonoses

## Abstract

In the present work, we describe the clinical–pathological case of an 11-month-old Border Collie dog, which was presented by its owner to a private veterinary clinic for the purpose of determining the diagnosis and subsequent therapy. The owner reports anamnestic data of abdominal enlargement, persistent apathy, fatigue, and vomiting. A complete examination of the patient was performed, consisting of clinical, hematological, and biochemical blood tests, X-ray, and USG examinations. Based on the findings, a probatory laparotomy was indicated, during which a large multi-lobular cystic irregular mass was detected, affecting the entire liver parenchyma, including macroscopic metastatic foci of the omentum and diaphragm. Due to the inoperable finding, the patient was humanely euthanized during the surgical procedure. Subsequently, an autopsy was performed with the collection of samples for histopathological and PCR examination of the tissue. Serological examination was also performed. The results confirmed a rare generalized form of alveococcosis (*Echinococcus multilocularis*) in the dog as an intermediate host.

## 1. Introduction

Echinococcosis can be characterized as a zoonotic cestodosis caused by metacestodes, larval stages of *Echinococcus* spp. [1]. Currently, eight species of tapeworms of the genus *Echinococcus* are known: Fox tapeworm (*Echinococcus multilocularis*) is found exclusively in the northern hemisphere [2]. The main endemic areas are southern Germany, Switzerland, central and eastern France, and western Austria. Dog tapeworm (*E. granulosus sensu lato* (s.l.)) is distributed worldwide. In Europe, it is mainly found in the east and south, as well as along the Mediterranean coast. This tapeworm is rare in Germany [3]. *E. granulosus* s.l. represents a species complex that currently includes five different species with different morphological, biological and genetic characteristics: *E. granulosus sensu stricto* (genotypes G1–G3), *E. felidis*, *E. equinus*, *E. ortleppi* and *E. canadensis* (genotypes G6/G7, G8 and G10). The species *E. vogeli* and *E. oligarthrus* are found only in Central and South America [4,5]. The adult tapeworm *Echinococcus granulosus* (dog tapeworm) reaches a size of 2–9 mm and in adulthood parasitizes in the intestine of dogs, wolves, and jackals and its larval cyst causes cystic echinococcosis of ruminants and humans. The adult tapeworm *Echinococcus multilocularis* (fox tapeworm) parasitizes in the intestines of foxes, wolves, and other carnivores. In adulthood it reaches a size of only 1.3–3.7 mm and its larval cyst causes alveolar echinococcosis of intermediate hosts [6].

*Echinococcus granulosus* has a typical two-host life cycle where the definitive hosts are dogs or other carnivores and the intermediate hosts are domestic and free-living herbivores, but also humans. The definitive host is infected by consuming cysts present in the organs of infected animals and by the feces of the definitive host, a large number of eggs are released into the environment. The definitive host of *E. multilocularis* are foxes, canines, and feline carnivores. The intermediate hosts are domestic mammals and, occasionally, humans. The last segment of the tapeworm contains a uterus filled with eggs, which contain the infectious larva. After separation, the segment is excreted with feces and the eggs are dispersed in the environment. In the intermediate host, the oncosphere most often penetrates the liver but also other organs where it forms a larval stage—*metacestode*. Contact with an infected animal, its feces or even forest fruits that have been contaminated with the feces of an infected individual poses a risk to humans. In the definitive host, the disease progresses without obvious clinical symptoms, or only very mildly (diarrhea, loss of appetite, abdominal pain) [7,8]. However, there are confirmed cases when a dog (under normal circumstances the definitive host) also plays the role of intermediate host, when cysts formation with internal organs affection was observed [9,10]. Clinical manifestations also differ depending on the cyst’s location. When localized in the lungs, we observe cough, breathing problems, or chest pain [11,12]. In alveolar echinococcosis, a large number of small alveoli are formed primarily in the liver, but they can subsequently spread to other organs. The initial clinical symptoms include abdominal pain, indigestion, jaundice, and increased blood pressure. In untreated patients, the disease can be fatal [13,14].

The present report describes a case of alveolar echinococcosis in 11-month-old dogs from Slovakia, confirmed by histological and molecular methods.

## 2. Results

### 2.1. Anamnesis

On 19 January 2024, a patient from Poltár district (dog, male, border collie breed, 11 months old, fully vaccinated according to European veterinary standards, the last antiparasitic treatment was at the age of 3 months by administering a drug with a combination of active compounds pyrantel (14.4 mg/kg)—praziquantel (5 mg/kg)—febantel (15 mg/kg)) was brought to a private veterinary clinic. The owner of the animal states as the basic clinical symptoms inappetence, loss of appetite, lethargy, vomiting, and abdominal enlargement, which the owner attributed to excessive feeding in the last period.

### 2.2. Clinical Examination

During the clinical examination, we determined the physiological values of breathing (22 breaths per minute) and pulse frequency (115 beats per minute) as well as body temperature (38.1 °C). By palpation, we detected a bilaterally significantly distended abdomen, which was stiff, difficult to palpate, but painless.

**Figure 1:**
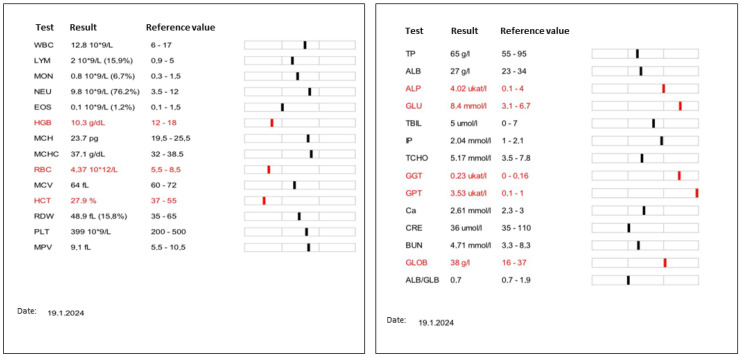
Complete results of laboratory assessment of hematological and biochemical parameters. Hematological examination indicates a mild degree of anemia (RBC 4.37  ×  10^12^/L; reference range [RR]: 5.5–8.5  ×  10^12^ /L; HGB 10.3 g/dL, RR: 12–18 g/dL; HCT 27.9%, RR: 37–55). Biochemical examination of blood serum indicates mild hyperglycemia (8.4 mmol/L, RR: 3.1–6.7 mmol/L) and hyperglobulinemia (38  g/L; RR: 16–37  mmol/L), increased activity of alkaline phosphatase (ALP, 4.02 µkat/L; RR: 0.1–4.0 µkat/L), gamma glutamyl transferase ([GGT], 0.23 µkat/L; RR: 0–0.16) and glutamic pyruvic transaminase ([GPT], 3.53 µkat/L; RR: 0.1–1 µkat/L). Other hematological and biochemistry parameters were within the physiological range.

Laboratory assessment included complete blood count, which was within normal limits with the exception of a mild anemia (RBC 4.37  ×  10^12^/L; reference range [RR]: 5.5–8.5  ×  10^12^ /L; HGB 10.3 g/dL, RR: 12–18 g/dL; HCT 27.9%, RR: 37–55). The serum bio-chemistry profile showed mild hyperglycemia (8.4 mmol/L, RR: 3.1–6.7 mmol/L) and hyperglobulinemia (38  g/L; RR: 16–37 mmol/L), increased activity of alkaline phosphatase (ALP, 4.02 µkat/L; RR: 0.1–4.0 µkat/L), gamma glutamyl transferase ([GGT], 0.23 µkat/L; RR: 0–0.16) and glutamic pyruvic transaminase ([GPT], 3.53 µkat/L; RR: 0.1–1 µkat/L). Enzyme activities of blood urea nitrogen ([BUN], 4.71 mmol/L; RR: 3.3–8.3 mmol/L), and creatinine (36 μmol/L; RR: 35–110 μmol/L) was within normal limits (Figure 1).

**Figure 2:**
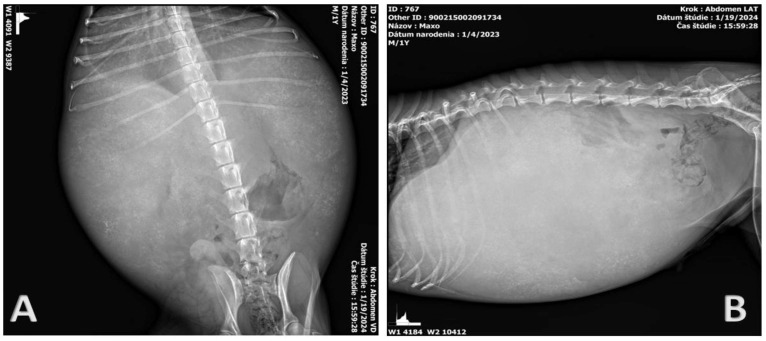
X-rays of the patient’s abdominal cavity in the dorsoventral (**A**) and latero-lateral (**B**) positions. The results of radiological examination indicate extensive ascites. The other structures cannot be precisely defined.

X-ray methods in the latero-lateral and ventro-dorsal position were used as an additional imaging diagnostic method. The results of the radiological examination indicate the presence of fluid in the abdominal cavity (Figure 2). The position or structure of the individual organs could not be accurately described. Due to the extensive presence of free fluid, it was not possible to perform a detailed ultrasonographic examination.

### 2.3. Probatory Laparotomy

**Figure 3:**
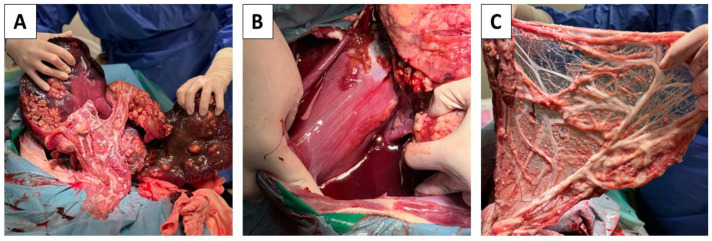
Pathologically altered abdominal organs. Altered liver parenchyma, hepatomegaly, light-colored proliferative foci present in the parenchyma of all lobes (**A**). Large amount of free fluid present in the abdominal cavity (**B**). Small cystic formations filled with transparent fluid found on the diaphragm and omentum (**B**,**C**).

Since the previous comprehensive examinations did not show or solve the situation, butorphanol (Butomidor, VetViva Richter GmbH, Welse, Austria) dosage 0.2 mg/kg and diazepam (Alzane, Laboratorios SYVA S.A.U., Avda, León, Spain) dosage 0.2 mg/kg were administered intravenously to sedate the patient before the operation. For the induction of anesthesia, propofol (Propofol MCT/LCT 1% Fresenius inj. inf., Fresenius Kabi Austria GmbH, Graz, Austria) in a dosage of 4mg/kg was used. Further prolongation of anesthesia was carried out by isoflurane (Isoflurin 1000 mg/g liq. inh., VETPHARMA ANIMAL HEALTH, S.L., Barcelona, Spain), dosage MAC 1.5–2.0%. The surgical site was prepared according to the principles of sterility and asepsis. After surgical opening of the abdominal cavity, a large amount of free fluid and hepatomegaly was observed (Figure 3A). Proliferative foci as well as small cystic formations filled with transparent fluid were observed in the parenchyma of the liver. Identical deposits were located on the omentum and diaphragm (Figure 3A–C). Due to the inoperable findings, euthanasia was indicated. The euthanasia was performed by continuous intravenous administration of combination *embutramide*, *mebezonii iodidum*, and *tetracaini hydrochloridum* (T61, Intervet International B.V., Boxmeer, Netherland) in dosage 0.5mL/kg).

### 2.4. Post-Mortem Findings

**Figure 4:**
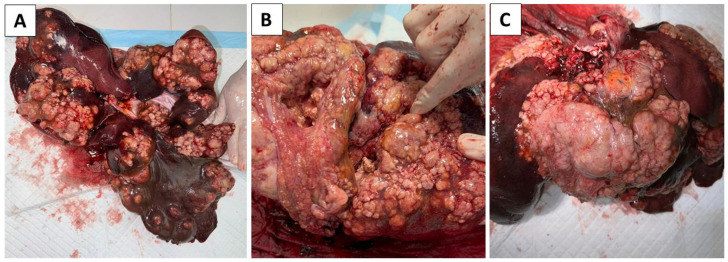
Pathological changes were found in the liver. All lobes were affected (**A**), showing tumorous changes (**B**) as well as the presence of small cystic formations (**A**–**C**).

Due to the suspicion of the presence of a zoonosis, all other measures were taken with stricter measures. The staff present worked in FFP2 masks, goggles, and two pairs of gloves. changes in the presence of proliferative foci and cystic formations were observed in all lobes of the liver (liver weight—5.65 kg) (Figure 4). Identical changes were observed in the omentum and diaphragm. Other organs were without the presence of pathological changes. Samples from all altered organs were collected, fixed in 10% buffered formalin and routinely processed for histopathological examination. Likewise, samples for PCR, microbiological and mycological examination were collected.

### 2.5. Histo-Pathological Study

**Figure 5:**
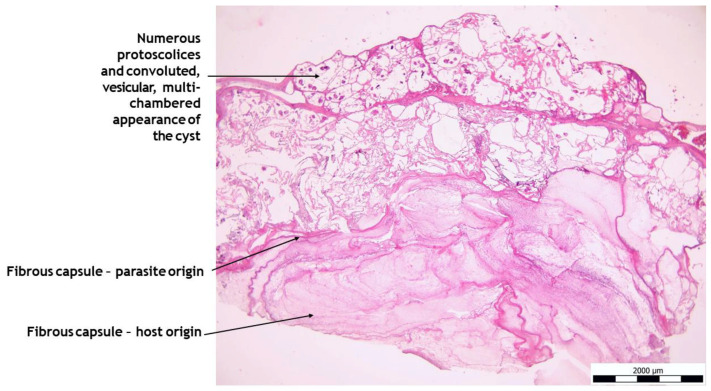
Hepatic manifestation of the larval stage of *E. multilocularis* in a dog liver resection specimen, HE, 20×.

**Figure 6:**
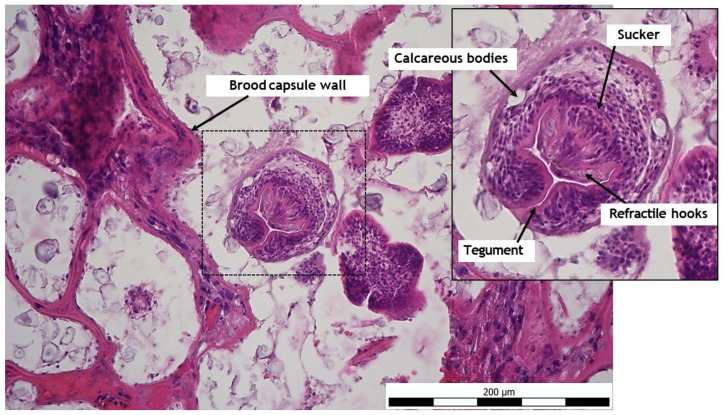
Hepatic manifestation of the larval stage of *E. multilocularis* in a dog liver with cross section of the protoscolice, HE, 200×.

Histologically, liver tissue samples showed ongoing necrosis of the liver parenchyma, including manifestation of the larval stage of *E. multilocularis* (Figure 5). Next, multiple hydatid cysts in the liver could be detected. Likewise, numerous protoscolices and convoluted, vesicular, multichambered appearance of the cyst were detected. The hydatid cysts were surrounded by a fibrous capsule and were lined with an acellular laminated layer that had a narrow, single-layered, nucleated cell layer towards the lumen, which often contained calcareous corpuscles. On cross-section of the protoscolices, concrete structures including tegument, refractile hooks, sucker, and calcareous body could be determined (Figure 6). Complete results of the histological study are presented in Supplementary Material S1.

### 2.6. Microbiology

Bacteriological study provided by culture method for the presence of aerobic and anaerobic strains of bacteria was negative. Likewise, mycological examination for the presence of mold was negative.

### 2.7. Parasitology

The patient’s blood serum was sent to a commercial laboratory for serological testing for the presence of antibodies against the causative agent *Echinococcus spp.* with the positive result; the value found was 18.60 NTU. A positive value is considered to be ≥11. Liver cyst material was analyzed for the presence of *E. granulosus* and *E. multilocularis* DNA.

In this study, we used multiplex real-time polymerase chain reaction (M-RT-PCR) targeting mitochondrial 12S rRNA gene of *E. granulosus* and *E. multilocularis* using Echi S (5′-TTTATGAATATTGTGACCCTGAGAT-3′) and Echi A (5′-GGTCTTAACTCAACTCATGGAG-3′) primers and three different probes; Anchor Ech (5′-GTTTGCCACCTCGATGTTGACTTAG-fluoroscein-3′), Granulosus (5′-LC640-CTAAGGTTTTGGTGTAGTAATTGATATTTT-phosphate-3′) and Multilocularis (5′-LC705-CTGTGATCTTGGTGTAGTAGTTGAGATT-phosphate-3′) (all from TIB MolBiol, Berlin, Germany) that will enable the diagnosis of CE and AE in same assay. During M-RTR-PCR, plasmids containing *E. granulosus* (GenBank: AF297617.1) and *E. multilocularis* (GenBank: NC_000928.2) mitochondrial 12S rRNA regions were used as positive controls according to methodology published by Can et al. [15]. Using the above method, we identified the presence of *E. multilocularis* in the sample.

## 3. Discussion

The presented clinical report of a dog infected with metacestodes *E. multilocularis* with developed symptoms of alveolar echinococcosis represents the second documented case in Slovakia. The first finding of alveolar echinococcosis in the Slovak Republic dates back to 2016 [16]. However, the first documented case of *E. multilocularis* in a dog in Slovakia was published in 2009 [16]. The described case comes from the Poltar district, which is close to an endemic district with a prevalence of *E. multilocularis* in red foxes (*Vulpes vulpes*), reaching 30–50%. Additionally, a human case of AE presented in a 2024 study was also described in the district [17]. Published findings suggest that there is a close connection between the occurrence of alveolar echinococcosis in humans and the prevalence of echinococcosis in free-living carnivores and dogs, as the number of AE cases in a given region increases every year. The reason for the increase in the incidence of AE also lies in the lifestyle of the inhabitants of the given region, who often visit forests to collect berries and mushrooms.

Among the surrounding countries, the occurrence of *E. multilocularis* has been confirmed in the Czech Republic, Austria, Poland, and Hungary [18,19,20,21]. *E. multilocularis* infection is one of the most dangerous zoonoses in the Northern Hemisphere and causes more human deaths than rabies in Europe [22]. This is also evidenced by published works on the occurrence of *E. multilocularis* in the countries of Europe, Russia, Asia, Canada, and the USA [23,24,25,26]. The main definitive hosts of *E. multilocularis* in the geographical conditions of Central Europe include the red fox (*Vulpes vulpes*), as well as the grey wolf (*Canis lupus*) or raccoon (*Procyon lotor*) [27,28]. The fox and its feces represent the most likely source of infection in the described case. The epidemiological investigation revealed that the dog grew up and lived on a sheep farm where the fox population was high and could have come into contact with their feces. The role of a domestic dog (*Canis lupus familiaris*) as an intermediate host of *E. multilocularis* with the development of AE and symptoms is rather rare, but similar cases have been described not only in Slovakia but also in Europe, the USA, and Canada [29,30,31,32]. When investigating the route of infection in a given case, the ingestion of food contaminated with infectious feces, or direct consumption of infectious feces with high eggs load, comes into consideration. A concurring view on the possible infection of the dog and its subsequent acting as an intermediate host is also described in the works of Zajac et al. and Kolapo et al. [31,32]. Clinical symptoms of alveolar echinococcosis in dogs as intermediate hosts depend on the extent of involvement of a specific organ and correlate with clinical symptoms of alveolar echinococcosis in other animal species and human. The most common involvement is of the liver, spleen, omentum, and lungs [33,34,35,36]. In our case, we observed abdominal dystonia, anorexia, lethargy, changes in biochemical (liver function related), and hematological (haematocrit, haemoglobin, red blood cells) parameters. Sometimes changes in hematological indicators of the white blood cell count, such as neutrophilia or lymphopenia, may be observed [37]. Imaging methods such as ultrasound, X-ray, computed tomography, or MRI also play a role in the diagnosis and differential diagnosis of AE in human and veterinary medicine [38,39]. In the diagnostics of this case, we used USG and X-ray to examine the abdominal cavity; however, the results could not be interpreted, since a detailed examination of abdominal cavity organs was not possible due to the large amount of fluid released. By probatory laparotomy, we found cystic, neoplastic changes in all lobes of the liver and omentum. Due to the surgically intractable inoperable condition and extensive liver damage, we proceeded to euthanize the patient and collect samples for molecular diagnostics and patho-histological examination. In molecular diagnostics we chose the multiplex real-time polymerase chain reaction (M-RT-PCR) method, which, in combination with histological examination, is becoming the gold standard in the diagnosis of tissue parasites [40,41].

All findings and facts published in the present report point to the zoonotic potential and to a strong connection between published cases of AE in humans and the incidence of echinococcosis in wild carnivores and domestic dogs not only in Slovakia but also in Europe. These findings could serve as a basis for education in the fight against AE in human patients, which should be based on compliance with the principles of hygiene in the collection and consumption of forest fruits, mushrooms, and medicinal plants.

## 4. Conclusions

Although free-living and domestic carnivores are considered definitive hosts in the life cycle of *E. multilocularis*, it is possible that under certain circumstances they may also act as aberrant intermediate hosts, as demonstrated by this case. For this reason, it is necessary to consider this possibility in the pre-diagnosis and differential diagnosis of gastrointestinal tract diseases, endocrine diseases with symptoms of abdominal distension and ascites, especially in animals and humans originating from endemic areas of echinococcosis.

## Data Availability

The data presented in this study are available upon request from the corresponding author.

## Supplementary Materials

The following supporting information can be downloaded at: https://www.mdpi.com/article/10.3390/pathogens14050450/s1, Figure S1: Hepaticmanifestation of *E. multilocularis*. Numerous protoscolices and convoluted, vesicular, multichambered appearance of the cyst, HE, 40×. Figure S2: Histological study of protoscolex of *E. multilocularis*, HE, 1000x.
