# Supplementary material for: Alveolar Echinococcosis in 11-Month-Old Dog—Clinical Case
Source: Pathogens. 2025 May 2;14(5):450. doi: 10.3390/pathogens14050450 (PMC12114745; doi:10.3390/pathogens14050450)

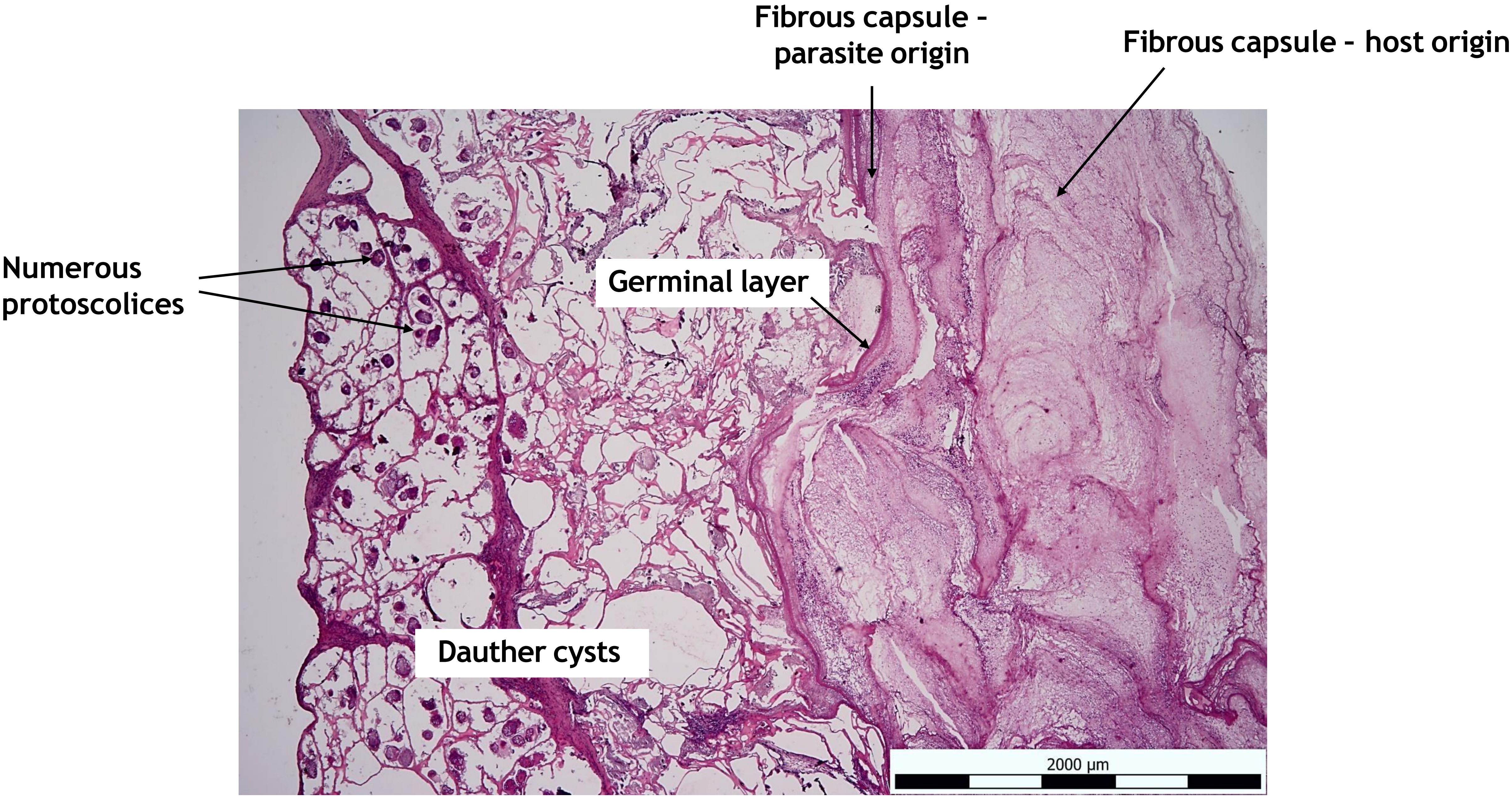

Figure S1: Hepatic manifestation of *E. multilocularis*. Numerous protoscolices and convoluted, vesicular, multi-chambered appearance of the cyst, HE, 40x

Figure S2: Histological study of protoscolex of *E. multilocularis*, HE, 1000x

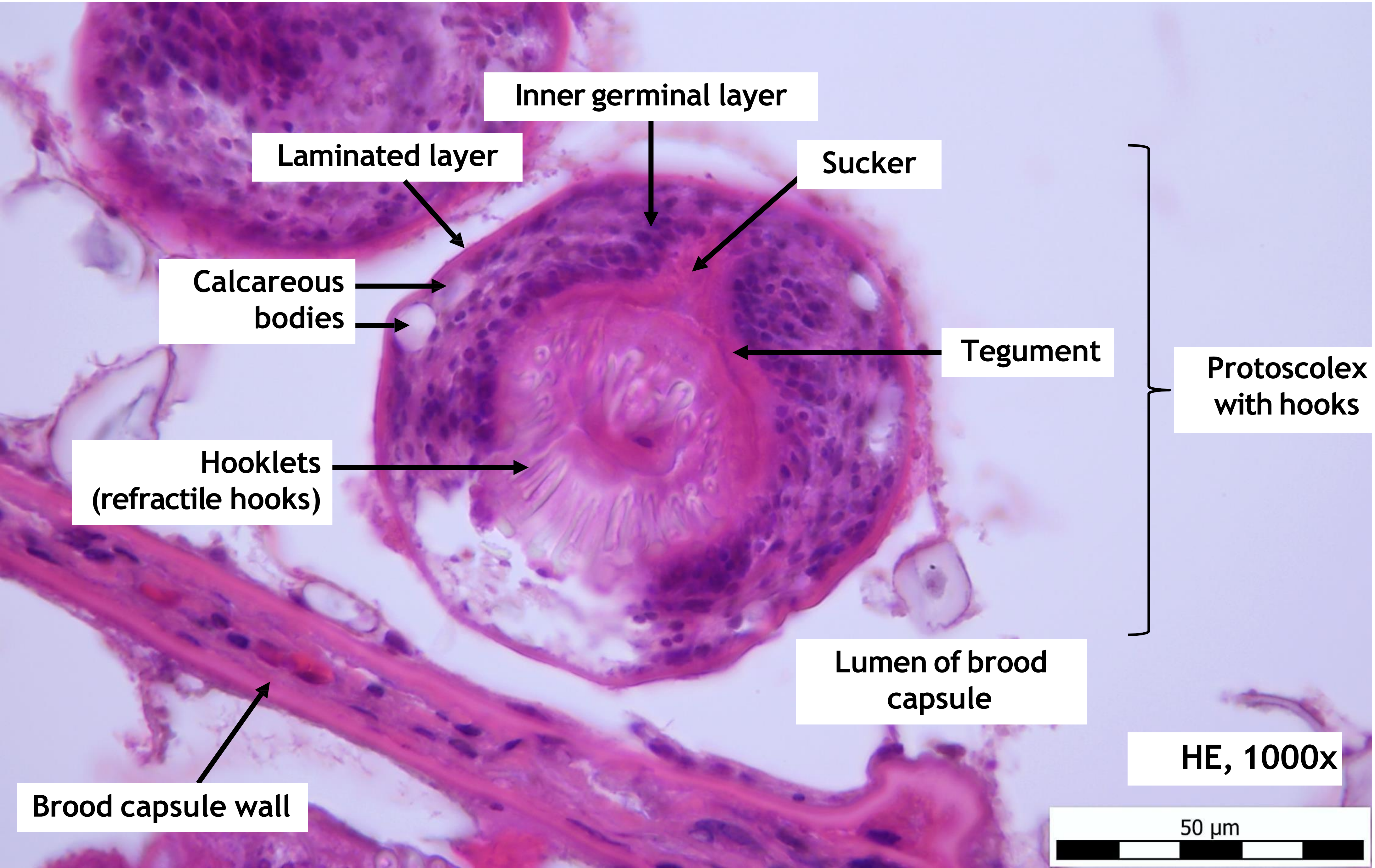

Supplement: Supplementary file 1 [file pathogens-14-00450-s001.zip › pathogens-3584142-supplementary.pdf]
